# Supplementary material for: Are carbon emissions trading and green financial instruments synergistic? -Comprehensive quantitative research based on content analysis
Source: PLoS One. 2024 Mar 7;19(3):e0298601. doi: 10.1371/journal.pone.0298601 (PMC10919601; doi:10.1371/journal.pone.0298601)
Supplement: S1 Data — (ZIP) [file pone.0298601.s001.zip › Data/地级市Z_F工作报告.zip!/╜¡╦╒/╠⌐╓▌╩╨/└┤╫╘╣1⁄2╓┌║┼í╛┬φ┐╦╩2╛▌═°í┐_2018.docx]

各位代表：

现在，我代表市人民政府向大会作工作报告，请予审议，并请市政协委员和其他列席人员提出意见。

一、2017年工作回顾

过去的一年，我们在中共泰州市委的正确领导下，坚持稳中求进工作总基调，自觉践行新发展理念，以供给侧结构性改革为主线，以“四个关键突破”为抓手，扎实推进“两聚一高”新实践，加快建设“强富美高”新泰州，较好地完成了市五届人大一次会议确定的各项目标任务。预计实现地区生产总值4740亿元，增长8.1%；一般公共预算收入344亿元，增长5%；固定资产投资3620亿元，增长14.3%；社会消费品零售总额1257亿元，增长12.4%；实际利用外资16亿美元，进出口总额125亿美元，外经营业额8.8亿美元，分别增长19%、20.4%、1.3%；城乡居民人均可支配收入分别达40069元、19469元，分别增长8.8%、9%；居民消费价格涨幅1.9%；城镇登记失业率1.82%。

突出创新引领，转型升级步伐加快。坚持以实体经济为基点推动创新，谋划建设长三角地区特色产业基地、区域产业科技创新高地，经济发展质态进一步提升。产业结构继续优化。工业经济稳中提质，规模以上工业增加值、利润分别增长9.4%、15%；工业投资增速持续保持全省领先，固定资产抵扣增长48.8%；工业一般纳税人开票销售5294.7亿元，增长20.8%；新增销售10亿元以上企业、利税过亿元企业各20家。建筑业实现产值3538亿元，新增特级资质企业4家。“一业牵引、三业主导、特色发展”的产业体系加快构建，大健康产业集聚发展试点方案获批，三大主导产业产值增长15%；推进“两化深度融合”，改造提升传统优势产业，实施企业智能化改造项目221个，新增工业机器人406台。服务业发展活力增强，实现增加值2240亿元，占地区生产总值比重提高0.6个百分点，新增省级生产性服务业集聚示范区3家；开展国家全域旅游示范区创建，成为国家中医药健康旅游示范区创建单位，全年接待游客2500万人次，实现旅游总收入320亿元；实施金融改革两年行动计划，组建市金控集团，建成产融综合服务中心、“1+10”产业投资基金体系和大健康股权融资撮合平台，新增新三板挂牌企业8家，医药高新区成为全国首个资本项目收入兑换便利化试点。科技创新成效突出。制定“1+4”政策体系，配套开展“十大工程、十大载体、十大平台、十大联盟”建设，突出抓好大院大所对接合作，高点谋划国际创新活力区发展。研发经费支出占地区生产总值比重2.55%，科技进步贡献率62%，高新技术产业产值占规模以上工业比重43%，获评全国创新驱动助力工程示范市。新认定市级以上高新技术企业200家，新增国家级科技企业孵化器1家、省级众创空间8家，大中型工业企业和规模以上高新技术企业研发机构建有率96%。发挥泰科易平台和技术专家巡诊团作用，新增发明专利授权700件、高新技术产品650个，获批省重大成果转化项目6个、研发计划项目20个。挂牌成立市招才局，出台“人才生态36条”，新增国家“千人计划”专家40名。市政府获中国质量奖提名奖，全国质量强市示范城市创建通过省级验收，品质城市评价指标体系成为省地方标准，主导和参与制（修）订国际标准9项，新创省名牌产品6个，新申报中国驰名商标4件。项目建设量质齐升。构建“一体两翼”招商格局，实施“2266”招商计划，新签约亿元以上产业项目1425个，其中总投资超过50亿元的16个、超过100亿元的6个，新批3000万美元以上外资项目89个。严格执行“五个一”督查推进机制，新开工亿元以上项目541个，其中10亿元以上项目39个，总投资110亿元的中冶新材料产业园开工建设；亿元以上项目竣工445个，完成产业投资3180亿元。推进开发区提档升级，医药高新区新落户生物医药及医疗器械企业近300家，中科院大化所生物医药创新研究院、国际遗传工程与生物技术中心等平台载体加快建设，药物安评中心投入运营；泰兴经济开发区、泰州港经济开发区进入省级开发区十强，海陵工业园获评中国产业园区成长力、创新力百强，姜堰经济开发区创成省级知识产权示范园区。海关、国检、海事、边检为开放开发作出积极贡献。

注重品质提升，城乡面貌不断改善。制订参与扬子江城市群建设行动方案，实施城建新提升新两年行动计划，促进城乡融合发展。中心城市功能环境加快提升。坚持规划引领，组织城市发展战略研究和第四轮城市总体规划修编。推进中心城区快速路网建设，东风路南段、永定路东段建成通车，G328海姜段完成桥梁桩基施工，S231北段、站前路、永定路西段开工建设。实施道路接通和“微循环”畅通工程，济川路、梅兰路及红旗大道东延等加快建设，太湖路、淮河路、公园路、老东谢路等建成通车。西客站片区改造进入扫尾阶段，体育公园、海陵东部片区改造等重点工程启动建设，人民医院、中医院新院建成投用，双河小学、初中基本竣工，科技馆、南山寺等文旅项目即将完工，凤城河景区提升总体规划启动编制。完成棚户区改造1.7万户、老旧小区“微整治”30个，新增停车位超过5000个，提档升级农贸市场44家。推进绿满泰州建设，凤凰绿带、祥泰（城西）公园、永定游园等相继竣工，凤凰河景观提升工程建成开放。改造老旧供水管网65公里，铺设改造污水管网146公里，引江河二期水系治理、第二城南污水处理厂扩建等项目基本完工。优化城市治理与服务，实现全国文明城市“二连冠”，创成省优秀管理城市。重大交通基础设施建设取得突破。北沿江高铁预可、可研及勘察设计项目招投标工作正式启动，盐泰锡常宜铁路设计时速调整为350公里，两条高铁均纳入省交通重点项目三年推进计划，常泰、张靖等4条过江通道前期工作全面启动。阜兴泰高速兴化至泰州段建成通车，京沪高速江广段扩容工程双向八车道全线贯通，高兴东高速列入新一轮全省高速公路规划。泰东河、川东港整治全面完工，新通扬运河航道整治有序推进。扬泰国际机场通过民航安全管理体系运行效能评估，泰州港集装箱吞吐量达32万标箱。农业农村现代化加快推进。国家现代农业示范区建设走在全国前列，获批创建国家农业可持续发展试验示范区；推广适度规模经营“桥头经验”，新增家庭农场743家、为农服务综合体58个，农业综合机械化率82%；创成国家级出口食品农产品质量安全示范区，兴化获批创建全国首家农产品电子商务标准化示范区。推进新型城镇化和城乡发展一体化，城镇化率提高到64%；完成10个特色小镇的规划编制和评审报批，黄桥、戴南创成国家级特色小镇；加强特色田园乡村建设，兴化千垛田园综合体成为国家级试点，缸顾乡东罗村等5个村成为省级试点；新创“强富美高”示范村60个，改造农村危房1800户，新改建农村公路313公里，泰兴创成全国“四好农村路”示范县。农村改革稳步推进，全面完成土地承包经营权确权登记颁证，“三资”管理信息平台上线运行，农村产权交易项目数和金额保持全省前列，政策性农业保险覆盖面进一步扩大，市供销社获批国家级联合社“三会”制度专项改革试点。

坚持生态优先，环境质量持续向好。践行绿水青山就是金山银山的理念，推进国家生态文明建设示范区创建，绿色发展成效显现。环境问题治理动真碰硬。扎实推进“263”专项行动，减少化工企业203家，完成省定减煤指标，新增生活垃圾分类投放设施近2万个，9条黑臭河道整治达标，畜禽养殖污染关停户数、治理率均居全省首位，挥发性有机物治理经验全省推广。实施“蓝天工程”、“碧水行动”，空气质量优良率超过省定指标，省控以上断面优于Ⅲ类水质比例居全省首位。抓好环保督察问题整改，开展环境风险隐患排查，完成117家重点企业环境安全达标建设。生态经济布局全面展开。明确“两片一带”总体功能布局，率先出台里下河生态经济示范区发展规划，姜堰创成全国首批生态文明建设示范区。大力发展绿色产业，实施节能改造项目102个，2家企业被工信部认定为“绿色工厂”，3个省级园区循环化改造示范点通过考核验收。严把新上项目环保门槛，推动产业结构绿色调整，单位地区生产总值能耗下降3.7%，主要污染物排放量削减完成年度目标任务。生态文明制度更趋完善。加强规划管控，守牢生态红线，全面划定永久基本农田。大力推行河长制，实现市县乡村全覆盖，落实饮用水源地保护制度。探索生态经济区差别化考核，试点范围扩大到35个镇街。健全环保信用体系，加强评价结果应用，参与评价的非国控企业数居全省首位。开展领导干部自然资源资产离任审计，实行最严格的生态环境保护制度。

增进民生福祉，社会事业全面进步。坚持以人民为中心的发展思想，制定基层基本公共服务功能配置标准及实施方案，着力保障和改善民生。群众获得感得到增强。制定实施“创业富民30条”，发放创业富民“三张券”2.2亿元，构建创业培训“1+5”体系，新增创业示范基地65家，创业人员、市场主体分别增长41.2%、48.2%。建立经济发展与扩大就业联动机制，建成人力资源服务产业园，新增城镇就业10万人。落实城乡居民基本医疗保险制度，开展生育保险与职工医疗保险合并试点，率先推行社保卡就医一卡通，城乡低保标准、养老保险待遇、医疗救助比例进一步提高。抓好新一轮脱贫攻坚，出台健康扶贫补充保险实施方案，推进“阳光扶贫”监管系统建设，50%的建档立卡低收入农户和经济薄弱村实现脱贫。新建城乡标准化社区居家养老服务中心146家，夕阳红中心即将投用。新开工各类保障性住房超过1万套，住房保障诚信体系建设经验全省推广。百姓幸福感得到提升。学前教育省优质园比例居全省首位，义务教育现代化学校创建步伐加快，高考本一、本二达线率分别高于全省7个、21个百分点，南京中医药大学泰州校区计划秋季招生。深化文化市场综合执法改革，建成基层综合性文化服务中心563家，市区发放文化惠民券1000万元。医药卫生体制改革纵深推进，国家中医药综合改革试验区、市区医联体建设进展有序，率先推行高值医用耗材集中采购，市级药物临床试验机构实现零突破，15分钟健康服务圈基本形成。举办铁人三项亚洲杯、国际半程马拉松等重大赛事，泰州健儿在全运会上再获佳绩。做好双拥优抚安置工作，保障退役士兵和优抚对象合法权益。民族宗教、社会科学、广播电视、妇女儿童、残疾人、史志档案工作取得新进步，统计、气象、防震、民防、台务、外事侨务及对口支援工作取得新成绩。居民安全感得到保障。深入开展大排查大整治，实施信访积案“双百攻坚”、打击“两抢一盗”、治理非法集资等专项行动，圆满完成十九大安保维稳任务，万人八类刑事案件发案率连续6年全省最低，进京非访量连续8年全省最少。推进综治网格整合和社会治理大数据平台建设，健全立体化、信息化社会治安防控体系，连续6年获评全省社会治安综合治理先进市。深化“七五”普法，开展“百日维薪”法律援助行动，建成12348公共法律服务平台。构建“双重预防”机制，强化重点行业领域监管，安全生产形势总体稳定。创新食品安全监管方式，推进食品快检机构建设，靖江创成全省食品安全示范城市。应急管理平台建成运行，应对突发事件能力进一步提升。

转变工作方式，自身建设得到加强。牢固树立“四个意识”，加快转变政府职能，努力建设人民满意政府。深化改革促发展。推进行政审批制度改革，市及高港、医药高新区行政审批局挂牌运行，“审改630”、“3550”取得阶段性成果，出台不见面审批实施方案和目录清单，率先推行证照分离改革，在全国首创大数据O2O审图云平台。推进事业单位分类改革，探索义务教育阶段教职工编制统筹调度，实行公立医院编制备案制管理。推进国资国企改革，制定实施5家国企改革方案，出台强化国资监管、深化薪酬制度改革等政策。推进财税体制改革，健全综合治税、联合办税机制，新增政府和社会资本合作入库项目11个，累计获省财政转贷置换政府债券资金467.3亿元。推进国土资源综合改革，创新经营性用地上市机制，建立工业用地综合评估和履约保证金制度，提高节约集约用地水平。推进审计管理改革，基本实现审计全覆盖。依法行政强规范。坚持科学民主依法决策，成立市决策咨询委员会，建立公平竞争审查制度，制定政府规章3件，政府决策行为进一步规范。落实行政首长出庭应诉制度，办结行政复议申请210件，法律顾问制度加快向基层和企事业单位覆盖，泰兴、医药高新区综合执法体制改革试点扎实推进。自觉接受人大法律监督、工作监督和政协民主监督，广泛听取各民主党派、工商联、无党派人士和社会各界意见，提请市人大常委会审议地方性法规3件，人大代表建议和政协提案办理满意率达99%。深化政务公开工作，在全省第三方评估中居设区市首位。勤政廉政提效能。扎实开展“大走访大落实”，定期组织“三服务”活动和企业家座谈会，充分发挥12345热线、市长信箱等平台作用，解决了一批基层和群众反映的突出问题。加快政务服务“一张网”建设，在全省首创政务云服务外包模式。构建“三重”督查推进机制，建成市党政督查系统。认真贯彻中央八项规定精神和省市委十项规定，坚持不懈整治“四风”，严格落实“一岗双责”，严肃查处违纪案件和不正之风，重拳整治政府投资项目招投标市场。推进“两学一做”学习教育常态化制度化，开展“对标找差、跨越赶超”实践活动，激发机关干部干事创业精气神。

各位代表，过去一年所取得的成绩，是中共泰州市委正确领导的结果，是市人大、市政协及社会各界关心支持的结果，是全市人民团结拼搏、奋发作为的结果。在此，我代表市人民政府，向全市广大干部群众，向全体人大代表、政协委员，向各民主党派、工商联、无党派人士、各人民团体和广大离退休老同志，向中央和省驻泰机构以及驻泰部队指战员、武警官兵和政法干警，向所有关心支持泰州发展的海内外朋友们，表示衷心的感谢！

我们也清醒地认识到，发展不充分仍然是最大的市情实际，在扬子江城市群中还处于奋力追赶的关键阶段，提升发展质量和效益任重道远；产业结构仍然偏重，工业经济主要还是依赖传统产业带动，培育战略性新兴产业、推动经济转型升级还要狠下功夫；项目质量仍需提高，基地型、龙头型项目数量偏少，项目建设的评价体系有待进一步完善；为企服务方式需要创新，服务形式内容与企业实际需求不够贴近，政策资源的集成度和可及性还需提升；中心城市首位度不高，整体开发和高效利用城市资源不够充分，运用市场机制建设和经营城市还要深入探索、大胆实践；沿江地区与黄桥老区、里下河地区发展仍不平衡，推进区域协调发展还要花更大气力；公共服务供给不足，优质教育资源短缺的矛盾仍较突出，医疗卫生面临“补短”与“促强”的双重压力，养老服务设施建设相对滞后，有限财力与民生刚性需求存在不小差距；城乡居民收入增长乏力，财产性收入比重偏低，富民增收迫切需要拓宽渠道；政府职能转变还不到位，庸懒散现象仍然存在，敷衍塞责、推诿扯皮等作风问题必须坚决纠正。

二、2018年工作任务

今年是贯彻党的十九大精神的开局之年，是改革开放40周年，是决胜全面建成小康社会、实施“十三五”规划承上启下的关键一年，做好今年政府工作具有十分重要的意义。

今年政府工作的总体要求是：全面贯彻党的十九大精神，以习近平新时代中国特色社会主义思想为指导，按照总书记视察江苏重要讲话要求和中央经济工作会议、省委十三届三次全会、市委五届四次全会部署安排，坚持稳中求进工作总基调，坚持新发展理念，坚持高质量发展，坚持系统化思维，统筹做好稳增长、促改革、调结构、惠民生、防风险各项工作，加快推动质量变革、效率变革、动力变革，深入推进“两聚一高”新实践，扎实推进“四个关键突破”取得更大成效，努力推进高水平全面建成小康社会取得过硬成果，积极进行基本现代化建设新探索，把“强富美高”新泰州建设不断推向前进。

今年全市国民经济和社会发展主要预期目标为：地区生产总值增长8%左右，一般公共预算收入增长6%左右，固定资产投资增长10%，社会消费品零售总额增长11%，城乡居民收入与经济同步增长，居民消费价格涨幅3%左右，城镇登记失业率控制在3%以内，研发经费支出占比2.6%，节能减排完成省定目标任务。

今年将重点抓好十个方面的工作：

1. 打造特色产业高地。集聚发展大健康产业。围绕构建“药医养食游”全产业链，实施“健康+产业”转型行动，以中国医药城为核心统筹市区健康产业资源，加快发展精准医疗、康复疗养、康养旅游、健康农业和健康食品产业，打响“健康城市”品牌。做大做强先进制造业。聚焦疫苗、诊断试剂、高端医疗器械、“两高”船型和节能环保船舶、动力电池和储能技术等细分领域，超前布局关键核心技术研发，大力发展具有自主知识产权的高端产品，推进重大集成创新和产品应用示范，力争三大主导产业产值增长12%以上。实施企业互联网融合提升和智能制造发展工程，抓好服务型制造集聚基地、示范企业和项目建设，培育制造业转型升级新动能。抢抓军民融合发展机遇，发挥军工配套产业优势，创建国家级军民融合示范基地。提升发展现代服务业。实施服务业提速发展六大行动，加快构建以高端商贸、创新服务、现代物流、全域旅游和电子商务为主导的产业体系，推动生产性服务业专业化高端化、生活性服务业精细化品质化，力争服务业增加值占地区生产总值比重提高1个百分点。不断壮大特色产业。突出汽车零部件、化工新材料、电子信息、食品加工等传统优势产业，着力补强产业链条、完善配套体系，形成规模集群效应。实施大数据发展规划，加快建设政务云、商务云、工业云等应用平台。推进国家全域旅游示范区创建，开展旅游风景廊道建设试点，促进文旅融合，丰富旅游业态。

2. 筑牢实体经济根基。转变引导方式。研究制定对产业发展有重大促进作用的技术标准体系，明确并逐步提高环保、降耗、质量、安全等指标，倒逼企业加快淘汰低端落后产能，积极发展新产品、新服务、新业态、新模式。分行业制定重点企业和高成长性企业培育计划，推动企业争创“行业首企”。引导中小微企业向“专精特新”方向发展，培育科技“小巨人”企业30家。增强内生动力。组织实施一批以科技创新为先导、市场需求为依托、国际标准为参照的重大项目，力争工业技改投入增长12%。加快品质泰州建设，开展质量提升行动，建立健全质量激励制度，创成全国质量强市示范城市。实施商标品牌战略，推进全国知名品牌示范区创建，新创省名牌产品8个、中国驰名商标4件。优化企业服务。发挥好产业投资基金、产融综合服务中心、差别化土地供应政策的作用，提高为企服务资源的集成度和可及性。扩大扶持企业政策惠及面，定期开展技术、人才、金融对接活动，让“三服务”各项举措更加贴近企业需求。构建亲清新型政商关系，依法保护企业家财产权、创新权和自主经营权，激发和保护企业家精神。

3. 厚植创新发展优势。全力推进企业创新。实施企业研发机构建设三年行动计划，建立高新技术企业培育库，新建省级以上企业研发机构20家，新认定市级以上高新技术企业200家。围绕主导产业和特色板块，组织实施一批前瞻性产业技术创新专项和重大科技成果转化专项，力争实施共性关键技术攻关和成果转化项目20个。深化产学研协同创新，加快产业技术创新联盟和高价值专利培育示范中心建设，新增发明专利授权700件。全力突破创新载体。围绕“一产业一高端研发平台”目标，加强与大院大所的对接合作，力争国科大泰州大健康产业研究院等一批创新载体投入运营。提升三大产业研究院和泰科易平台功能，推进各类科技孵化器、育成中心和离岸孵化基地建设，在应用研究、集成创新、转移转化上务求突破。支持医药高新区聚力“三个一批”打造创新发展的先导区、引领区、示范区，推动有条件的园区创建省级高新区，建立市级高新区创新驱动发展综合评价指标体系。全力营造创新生态。推动“1+4”系列政策落实落地，打造区域创新生态示范区。大力实施创新人才集聚工程，新引进高层次创新创业人才200名。强化科技金融体系建设，设立统一的风险补偿资金池，支持科技型企业多渠道融资。加强科技咨询、技术评估、专利代理等机构培育，提升科技服务业发展水平。

4. 构建全面开放格局。持续强化项目建设。实施“236”招商计划，办好国际医药博览会、新能源峰会，发挥各类商会、行业协会的桥梁作用，压实市（区）和园区的主体责任，确保招商引资力度不减、氛围更浓。全力招引科技含量和财税贡献高、投资体量大、辐射带动力强的优质高端项目，促进招商引资向招大引优转变。严格执行督查推进机制，全程跟踪项目建设进度，确保实现“854”目标，力争实际利用外资16亿美元、产业投资增长11%。调整优化考评导向，重点考核签约落户率、设备投资额、投资强度、单体规模和投入产出比，提高项目落地的实际成效。推动园区改革创新。按照“一特三提升”的要求，引导省级以上开发区突出主业、二次创业、争先进位，加快国际合作步伐，形成错位竞争优势。持续深化园区管理体制机制改革，以“简政、精兵、强服务”为重点加快“去行政化”。推动医药高新区围绕“十年三步走、力争双一流”目标，加快向现代产业园区转型，力争在全国高新区排名明显提升。转变外贸发展方式。加强外贸转型升级示范基地建设，提升生物制药、新能源、船舶基地专业化水平，支持泰兴创建国家化工新材料基地。积极打造医药、检验检测等服务外包品牌，促进跨境电子商务、市场采购贸易、外贸综合服务健康发展。深化国家药品进口口岸城市、靖江保税物流园创建，扩大先进技术设备、关键零部件和优质消费品进口。加快“走出去”步伐。主动融入“一带一路”建设，以建筑、化工、冶金、光伏、船舶等行业为重点，积极开展面向沿线国家的贸易促进活动，支持有实力的企业建立境外贸易、投融资和生产服务网络。

5. 力促改革落地见效。深化供给侧结构性改革。坚持用市场化、法治化手段破除无效供给，确保完成去产能目标任务。坚持房子是用来住的、不是用来炒的定位，加快建立多主体供给、多渠道保障、租购并举的住房制度，促进房地产市场平稳健康发展。推进百企上市（挂牌）行动计划，非金融企业直接融资占比提高到14%，守住不发生系统性金融风险的底线。实施降低实体经济成本十大专项行动，全年直接降低企业成本50亿元。深化行政管理体制改革。统筹实施相对集中行政许可权、“3550”、综合执法和“互联网+政务服务”、不见面审批等改革举措，实现各项改革集成配套、协同推进。全面开展承担行政职能事业单位改革，探索建立事业单位公益指数评价体系，推进政事分开、事企分开、管办分离。深化金融改革。突出支持产业转型升级这个关键，构建特色产业全生命周期的金融服务机制，重点抓好市金控集团高效运营、产融综合服务中心全面运行、金融服务区加快运转，扎实推进泰州农商行转型发展和法人证券公司筹建。深化国资国企改革。改革国有资本授权经营体制，提升国有企业资本实力、业务规模和信用等级，健全公司法人治理结构，促进国有资产保值增值。深化财税体制改革。加快建立现代财政预算和绩效管理制度，落实税制改革和减税降费政策。

6. 提升中心城市能级。完善规划体系。深化第四轮城市总体规划修编，加快区域交通、空间特色、民生设施、生态建设等方面的专项规划编制，推进中心城区控制性详规“一张图”整合。加快新城崛起。坚持经营城市理念，加强对发展定位、主体功能、产业及空间布局等重大问题的研究谋划，高标准、高品质建设新城。扎实抓好国际创新活力区规划建设，完成总体规划编制，制定先导区控制性详规、重点地区城市设计、重大项目修建性详规和相关专项规划，启动重点功能板块建设。深化海军文化园规划研究，加快实施海军诞生地旧址提升工程。全面推进体育公园建设，完成体育场、体育馆、游泳馆和健身馆基础工程。实施人民医院新院、中心血站、急救中心搬迁，开工建设妇幼保健院、第五人民医院。焕发老城活力。推进“城市双修”，更新整治“三河三街区”。抓好涵东历史文化街区和历史地段保护性修复，加强笔颖楼、宫氏住宅、万字会等历史遗存修缮。开展凤城河景区提升七大行动，环河慢行步道、桃园提升工程基本竣工。实施棚户区改造新三年计划，继续抓好老旧小区“微整治”。深入治理老城水系，采取“一河一策”整治黑臭水体，基本消灭建成区内黑臭河道。提高城市治理与服务水平。优化城市商业发展布局，完善提升三大商圈功能，逐步形成核心商圈为引领、专业市场为支撑、社区商业为补充的商贸体系，完成市区农贸市场改造升级。强化供水、雨污分流、排水防涝等设施改造，建成污水处理设施监管系统。加强绿满泰州建设，继续实施海陵西北片区生态修复，中心城区新增绿地400万平方米。推进智慧城市、海绵城市和地下综合管廊建设，促进城市管理由数字化向智能化升级。推进交通基础设施建设。全力争取北沿江高铁先导段在泰州开工建设，加快盐泰锡常宜铁路前期工作，力争常泰、张靖过江通道明年开工建设。积极开展沪陕高速广陵至平潮段扩容、高兴东高速前期工作，力争启动实施阜兴泰高速二期。围绕形成“四纵四横、井字放射”快速路网，加快S231北段、永定路西段和站前路改造，确保G328海姜段建成通车。推进京泰路南延、青年路北延等主次干道建设，完成鼓楼路南延、春晖路北延、运河路接通。改造提升主要出入口片区环境景观，完成交通干线沿线环境综合整治。做好实施通南地区引排工程前期工作，基本完成新通扬运河航道工程。优化扬泰国际机场航线航班，提升综合保障能力。加快港口名城建设，推进沿江港口一体化发展，深化泰州港与国内外著名港口的战略合作。

7. 实施乡村振兴战略。优化新型城镇体系。支持靖江、泰兴建设创新发展先导区、融入苏南先行区，支持兴化探索走好生态环保、富有特色、致富百姓的发展新路。完善9个小城市产业、生态和公共服务功能，促进农民就地就近城镇化。引导特色小镇健康发展，统筹抓好特色产业培育、高端要素集聚、承载功能提升等工作。推进特色田园乡村建设。构建“1+9+10”联动建设试点格局，尽快打造亮点、形成示范。建设美丽宜居乡村，建成省级绿化示范村45个。注重保护传统风貌、文化遗存和乡村肌理，启动编纂名镇名村志。因地制宜推动“厕所革命”，新增无害化卫生户厕11200座，逐步让农村群众用上卫生的厕所。完善农村生活污水处理、垃圾收运处置基础设施，整治县乡河道492条。继续推进“四好农村路”建设，力争实现村村通公交。深化农业供给侧结构性改革。推进国家农业可持续发展试验示范区建设，落实粮食安全市长责任制。加强智慧农业示范园区创建，整体推进高标准农田建设。实施农产品品牌建设三年行动计划，培大育强特色农产品电商平台。构建现代农业生产经营体系，培育以家庭农场为主的新型农业经营主体，发展多种形式适度规模经营。巩固完善农村基本经营制度，推动承包地“三权分置”改革，建立健全土地承包经营权设立变更登记制度；深化农村集体产权制度改革，扩大集体经营性资产改革试点范围。

8. 加强生态文明建设。探索生态经济发展模式。按照“两片一带”总体功能布局，推动生态资源转化为经济资源、生态优势转化为发展优势，致力打造全省生态经济示范区。开展企业绿色制造技术装备应用示范，推动生态工业示范园区、循环化改造示范试点园区创建，实施节能改造项目100个，力争新增“绿色工厂”2家。培育壮大生态农业、休闲旅游、乡村民宿等业态，建成市级休闲观光农业示范村10家。强化生态环境保护治理。继续推进“263”专项行动，认真抓好化工行业专项整治、畜禽养殖污染综合治理、饮用水源地达标建设、生活垃圾分类处置等重点任务。全力打好污染防治攻坚战，突出治气、治水、治土，推动环境质量持续好转，空气质量优良率70%以上，城镇污水处理率88%以上，重点水功能区水质达标率79%以上，创成国家水生态文明城市。大力实施湿地修复工程，开展里下河国家湿地公园试点。启动国土绿化行动，新增成片造林1.3万亩、完成森林抚育5万亩。严格保护耕地，坚决惩处破坏永久基本农田行为。树立生态优先、绿色发展导向。深化生态文明建设示范区创建，力争海陵创成国家级示范区。完善网格化环境监管体系，推行村居“两个委员”工作机制，将环保“触角”延伸到最基层。加强环保行政执法与刑事司法衔接联动，对各类环境违法行为零容忍。实行生态文明差别化考核，探索生态资本经营机制，提升发展的生态底色、生态价值、生态优势。

9. 推动文化繁荣发展。完善公共文化服务体系。优化配置城乡文化资源，加快基层综合性文化服务中心和城市书房建设。发挥好文化惠民券作用，精心组织纪念改革开放40周年和百团千场、百姓系列活动，提高群众性文化活动的受众率和参与率。完善文化管理体制，深化文艺院团改革，推行公共文化设施社会化运营试点。做强特色文化品牌。办好水城水乡国际旅游节、梅兰芳艺术节，放大红色文化、吉祥文化、盐税文化等品牌效应。策划梅派特色题材创作，筹建里下河文学馆，提升微电影原创力。加强名家大师和高水平创作人才培养，壮大文艺工作者队伍。发展现代文化产业。按照“1133”产业布局，加快重点园区、项目和特色街区建设，引导文化资源要素向优势企业适度集中，积极发展数字传媒、工业设计、动漫游戏等文创产业。深化全国文明城市创建。弘扬社会主义核心价值观，抓好日常化、具象化、生活化传播，用更多正能量温暖每个人的精神家园。深入实施公民道德建设工程，推进诚信建设和志愿服务制度化，提高全社会文明程度。深化群众性精神文明创建活动，让城市生活的基本准则、现代社会的文明规范深入寻常百姓家。

10. 做好富民惠民文章。促进城乡居民增收。坚持把就业作为民生之本，实施重点群体免费职业培训行动和蓝领精英培育工程，健全企业职工工资宏观调控和工资集体协商制度，使更多的劳动者掌握一技之长、有份稳定收入。落实好“创业富民30条”，继续开展“创富泰州”系列活动，让创业的舞台更宽广、致富的道路更通畅，力争新增创业5万人、带动就业8万人。坚决打好脱贫攻坚战，与困难群众同甘苦、齐奋斗、共打拼，确保75%的建档立卡低收入农户和经济薄弱村实现脱贫。将老百姓的安危冷暖时刻放在心上，重视解决部分失地农民未进保的问题，健全低保对象和标准动态管理机制，加快建设社会救助一体化平台。扩大主要险种参保覆盖面，加强基本医疗保险、大病保险、医疗救助的有效衔接，启动机关事业单位职业年金征缴。提高公共服务水平。优先发展教育事业，制定实施幼儿教育优质普惠发展三年计划，完善学前教育设施布局，加大公办园建设力度，强化幼儿园安全监管，为孩子们快乐成长营造良好环境；促进义务教育城乡一体化发展，提升普通高中特色内涵发展水平，完善现代职业教育体系，推动高水平大学设立泰州校区。加强健康泰州建设，逐级分片组建纵向医联体，提高基层医疗卫生机构服务能力，推进分级诊疗和家庭医生签约服务，抓好爱国卫生、疾病预防控制和残疾人康复工作，广泛开展全民健身活动。积极应对人口老龄化，搭建养老服务信息平台，推行养老服务“三张券”，城乡标准化社区居家养老服务中心覆盖率分别达60%、30%。创建省级公交优先示范城市，推广应用新能源汽车，市区新增停车位5000个。认真做好第四次全国经济普查工作。推动社会治理创新。全面实施网格化治理机制创新工程，加快建设社会治理大数据平台，积极推动工作重心下移。建立健全预测预警预防体系，加强网络安全与信息化工作机构建设，提高社会治安“打防管控”信息化、智能化水平。开展金融风险、市场秩序、治安隐患、执行难等专项整治，争创全省法治城市创建工作先进市、全国“七五”普法中期先进市，夯实社会稳定基本面。健全公共安全体系，实施食品安全战略，坚决遏制重特大安全事故，提升防灾减灾救灾能力，当好人民群众生命财产安全的捍卫者、守护者。

三、加强政府自身建设

打铁必须自身硬。我们要以党的十九大精神为指引，扎实开展“不忘初心、牢记使命”主题教育，牢固树立以人民为中心的发展思想，立足新时代，找准新坐标，以政府工作的新气象新作为，推进“四个关键突破”取得更大成效。

更为务实地做好政府工作。从讲政治的高度狠抓落实，不断增强“四个意识”，始终与以习近平同志为核心的党中央保持一致，坚决把省委、省政府和市委决策部署落到实处。贯彻新发展理念求真务实，紧紧围绕“四个高质量”的发展要求，以低调务实不张扬的作风抓深化、抓拓展、抓提升，致力解决发展不平衡不充分的问题。坚持系统化思维谋定后动，正确处理好整体与局部、当前与长远、力度与节奏的关系，决不为眼前利益、局部利益而搞一些短期行为和形象工程。

更有担当地做好政府工作。必须为改革发展勇担当，树立和坚持崇尚实干、勇于担责的鲜明导向，工作中允许失误、宽容失败，但决不允许“混日子”、做“太平官”。必须为创新创业勇担当，坚决把与创新发展的形势要求不相适应的工作方式“转过来”，把制约创业创富的制度性障碍“清除掉”，最大程度保障市场主体的充分自由和发展自主。必须为百姓幸福勇担当，既尽力而为，又量力而行，把为老百姓造福的事情真正办好办实，推动政府工作与民生需求“无缝对接”，着力满足人民群众对美好生活的新期待。

更高效率地做好政府工作。严格遵循重大行政决策法定程序，分级负责、主动决策、敢于拍板，防止“拖而不议、议而不决”，提升决策质量和效率。弘扬雷厉风行、马上就办的工作作风，以“钉钉子”精神抓好十项重点工程和三十项重点工作，对表面上应承、背地里拖延的消极怠工加强督查问责，提高执行效率。运用互联网技术和信息化手段，推进政府信息资源整合和数据共享，提升政务服务的便捷性和精准性，用服务效率激发市场活力和社会创造力。

更加廉洁地做好政府工作。坚持依法行政，善用法治思维和法治方式推动发展，将政府工作全面纳入法治轨道。加大政务公开力度，自觉接受人大法律监督、工作监督和政协民主监督，更加重视审计监督、群众监督和舆论监督，让权力在阳光下透明运行。高标准贯彻中央八项规定实施细则和省市委具体办法，着力整治“文山会海”，坚决防止“四风”问题反弹回潮。加强政府系统惩治和预防腐败体系建设，从严落实主体责任，全面履行“一岗双责”，严肃查处腐败行为，促进干部清正、政府清廉、政治清明。

各位代表，新时代赋予新使命，新时代开辟新征程，新时代要有新作为。让我们更加紧密地团结在以习近平同志为核心的党中央周围，在省委、省政府和市委的坚强领导下，不忘初心扎实工作，牢记使命开拓进取，为高水平全面建成小康社会和高起点建设扬子江城市群中部崛起城市而不懈奋斗！
